# Valorization of *Ginkgo biloba* Leaf Powder as a Substrate in King Oyster Mushroom (*Pleurotus eryngii*) Cultivation

**DOI:** 10.3390/life14050639

**Published:** 2024-05-17

**Authors:** Haikang Li, Peng Liu, Zihao Li, Congtao Xu, Jinlong Pan, Yi Zhou, Qingxiu Hu, Suyue Zheng, Yajie Zou

**Affiliations:** 1 State Key Laboratory of Efficient Utilization of Arid and Semi-Arid Arable Land in Northern China, Beijing 100081, China; lihaikang97@163.com (H.L.); liupeng8681@163.com (P.L.); snowglorm@163.com (Z.L.); xct19980909@163.com (C.X.); y962343787013@163.com (J.P.); zzzzzhouyi@163.com (Y.Z.); huqingxiu@caas.cn (Q.H.); 2 Institute of Agricultural Resources and Regional Planning, Chinese Academy of Agricultural Sciences, Beijing 100081, China; 3 Department of Gardens and Ecological Engineering, Hebei University of Engineering, Handan 056107, China; zhengsuyue@hebeu.edu.cn

**Keywords:** *Pleurotus eryngii*, nutritional value, *Ginkgo biloba* leaf powder, biological efficiency, substrate

## Abstract

*Ginkgo biloba* is widely planted as a colorful foliage tree, and its leaf can be used as a biomass energy source, but it has been underutilized for a long time. The aim of this study was to investigate the potential of garden waste as a substrate component in the cultivation process of the king oyster mushroom (*Pleurotus eryngii*), with the goal of enhancing both the yield of *P. eryngii* and the efficiency of energy use. The percentages of *G. biloba* leaf powder in the substrate were 10.5% and 21% to replace sawdust or sugarcane bagasse in a typical substrate. A substrate formulation that could completely replace sawdust and sugarcane bagasse was selected by analyzing mycelial growth rate, days of production, fruiting body length, biological efficiency, yield, stipe thickness, pileus diameter and laccase activity. The results showed that Y1 (treatment with 21% *G. biloba* leaf powder and sugarcane bagasse) had the highest yield (303.1 ± 31.9 g), which was higher than that of CK (control) (259.3 ± 37.4 g). The crude fiber content of the samples grown on substrate Y1 (as 7.43%) was higher than CK (7.37%). In addition, *P. eryngii* grown on substrate Y1 had the highest laccase activity for the complete colonization of the mycelium. Thus, these findings suggest that *G. biloba* leaf powder represents a viable and economical supplement for enhancing both the yield and quality of *P. eryngii*.

## 1. Introduction

Landscape waste typically includes grass clippings, leaves, bark, flowers, branches, and other woody materials [1]. *G. biloba*, as one of the most excellent and colorful types of foliage, will wither and drop its leaves after autumn, creating a large amount of garden waste [2]. Countries such as the United States, the United Kingdom and Japan have also promoted the composting of garden waste accordingly [3,4,5], but improper treatment will cause problems such as fire and environmental pollution [6]. *G. biloba* is rich in lignin, cellulose and hemicellulose, which are precisely the nutrients needed for edible mushrooms [7,8,9,10], making it a potential resource for mushroom cultivation.

In nature, white rot fungi are powerful lignocellulose decomposers because they are able to secrete a complete system of extracellular lignocellulose-degrading enzymes that act synergistically to facilitate lignocellulose utilisation [11]. *P. eryngii*, a vital white rot fungus, also known as the king oyster mushroom, is rich in polysaccharides, proteins, fiber, vitamins and minerals [12,13,14], and it produces secondary metabolites, such as ergosterol and lanosterol, which have been shown to reduce cholesterol levels in the human body and enhance the immune system [15]. According to the statistics from the China Edible Fungi Association, 1.52 × 10^6^ tons of *P. eryngii* have been cultivated in China in 2022. Consumers are increasingly acknowledging the role of this product as a functional food, attributed to its bioactive components that offer potential health benefits [16]. As the market demand for *P. eryngii* escalates, its cultivation is gaining paramount importance.

In China, the substrate for commercial production of *P. eryngii* include large quantities of sawdust and sugarcane bagasse [17,18], supplemented with other materials. Sawdust resources are restricted by forest protection policies, and the “mushroom–forest conflict” is prominent, which has become an important bottleneck affecting the edible mushroom industry benefits and restricting the sustainable development of the industry. Recently, a variety of garden wastes have been used for cultivation of *P. eryngii*, including *Phragmites australis* [19], umbrella plant (*Cyperus alternifolius*) [20], bulrush stalks [21], and burma reed (*Neyraudia reynaudiana*) [22]. Therefore, there is an urgent need to encourage the extensive use of garden waste for the rapid development of the *P. eryngii* cultivation industry. The development of garden waste resources to replace traditional cultivation raw materials has become imminent.

In this study, *G. biloba* leaf powder was used as a substrate for cultivating *P. eryngii*, and the feasibility of replacing sawdust or sugarcane bagasse raw materials with *G. biloba* leaf powder was also discussed. In order to achieve high yield of *P. eryngii*, the production cost of *P. eryngii* cultivation enterprises was effectively reduced, and energy saving and efficiency were achieved. In addition, the effect of the substrate on nutrient accumulation was estimated in this study. The results of this study promote the effective utilisation of *G. biloba* leaf waste resources, and *P. eryngii* cultivation can use *G. biloba* leaf powder as an alternative substrate, thus increasing *P. eryngii* yields and farmers’ incomes.

## 2. Materials and Methods

### 2.1. Inoculum Source and Spawn Preparation

*P. eryngii* (ACCC52611) was obtained from the Agricultural Culture Collection of China (ACCC), Institute of Agricultural Resources and Regional Planning, Chinese Academy of Agricultural Science. Mycelia were grown on potato dextrose agar (Becton, NJ, USA, PDA). They were grown in the dark at 25 °C for 10 days until most of the plate surface was covered with mycelia.

### 2.2. Substrate Preparation

*G. biloba* leaf originates from Haidian District, Beijing. Substrate particles were obtained by using a grinder (XL-60C). The grinder was purchased from HangZhou XuZhong Food Machinery Co., Ltd. (Hangzhou, China). Sugarcane bagasse was purchased from sugar mills and other materials from local markets. All materials are less than 0.5 cm in size and can be packaged in polypropylene bags. Table 1 provides the substrates that were used in this research. The control was the commonly used substrate of 21% sawdust and 21% sugarcane bagasse [23,24]. In substrate Y1 and Y2, *G. biloba* leaf powder replaced sawdust and sugarcane bagasse. In Y3 and Y4, *G. biloba* leaf powder replaced half of the sawdust and sugarcane bagasse. All treatments contained 4.2% cottonseed hull, 18.4% wheat bran, 18.4% ground corncobs, 6.8% maize powder, 8.2% soybean meal, 1% lime and 1% gypsum. These ingredients were thoroughly dry mixed, then mixed with tap water to give a final water content of 65% [25]. Subsequent preparation of the substrate was carried out according to Zhou et al. [9]. The sawdust was composted outdoors for 6 months to allow it to weather before being incorporated into the substrate. All other ingredients were not pre-treated before being added to the substrate. The loss of ignition and Kjeldahl methods were used to estimate the carbon (C) and nitrogen (N) content, respectively. The C:N ratio was determined for each substrate as previously described [26].

### 2.3. Assay for the Growth Rate

Mycelial growth rates were determined on all substrate combinations using linear growth methods as previously described [27,28]. Each combination of substrate was separately placed in glass tubes (320 mm long and 30 mm in diameter) with a density of approximately 0.8 g cm^−3^. A 5 mm diameter mycelial disc was used to inoculate the samples by placing it on top of the tube. The tubes were then sealed with sterile rubber stoppers and incubated at 25 ± 1 °C. After 5, 10, 14, 18, 22, 26 and 30 days of inoculation, the position where the mycelia had grown in the glass tubes was detected and the length of mycelial spread during the detection interval was calculated, and then the daily growth rate of the mycelia was obtained by dividing the length by the interval time. The rate of mycelial growth spread was assessed by observing the time of spawning (number of days that mycelial growth filled the substrate). This experiment was evaluated in five replicates for each group of substrates.

### 2.4. Spawning and Fruiting Body

*P. eryngii* was cultivated as described by Zhang [28] with minor modifications. For inoculation, the stick strain was prepared using polypropylene bags and the sticks were made of broadleaf tree wood with a mass of 1000 g. The sticks were soaked in 2% lime for 48–72 h until the sticks were completely saturated and then the excess moisture in the sticks was controlled. A mixture of wheat bran (50%) and maize flour (50%) was applied to the sticks. The sticks were then placed parallel to each other in the bag and the spaces between the sticks were filled with the control as described in Section 2.2. The sticks were then inoculated into sterilized culture bags. The strains were grown for 30–35 days at 24 ± 1 °C and 60% relative humidity in a light protected environment until the mycelium was completely covered. Incubation was continued under the same conditions for 5–7 days to allow the mushrooms to reach physiological maturity. The next step was to transfer the mushrooms to mushroom chambers for 2 days of acclimatization. The temperature of the mushroom chamber was 20 ± 2 °C, the relative humidity was 70–80%, and the CO_2_ concentration was 500–1000 ppm. The temperature of the mushroom chamber was set at 11–14 °C, with a white/black light cycle of 12 h, and a light level of 1500–2000 lux. The relative humidity was about 90% and the CO_2_ concentration was 1000–2000 ppm for induction of progenitor differentiation and fruiting body development. When mushrooms are in the seedling stage, adjusting the temperature to 10–13 °C and a CO_2_ concentration of 7000–8000 ppm promotes growth of the stipes. The agronomic characteristics of the fruiting body were determined using vernier calipers. The diameter and length of the stem and the diameter of the pileus were measured in cm. A wheel of fruiting body was produced, harvested and measured, which is consistent with the commercial cultivation practices of *P. eryngii* in China. The fruiting period lasted 18–20 days. The fresh yield of substrate was weighed, and 30 replicates were obtained after removing the extremes. The fresh yield of the substrate was divided by the dry substrate in each bag to calculate the percentage of bioefficiency (BE) [29].

### 2.5. Enzymatic Activity Assay

Mycelia, complete mycelial colonization, young mushroom stage and fruiting body stage cultivation substrate were taken for 250 g laccase activity assay, respectively. At the same time, *P. eryngii* mycelia were isolated from the cultivation substrate by tweezers, washed with sterile water to remove the residual medium and then rapidly frozen with liquid nitrogen and stored at −80 °C for spare use. A total of 5 g of *P. eryngii* cultivation substrate at different stages of growth and development was taken in 250 mL triangular vials, and 50 mL of 0.15 mol·L^−1^ NaCl solution was soaked. NaCl solution, centrifuged at 4 °C for 15 min, was filtered through filter paper, and the method of Bourbonnais et al. [30] was used to determine the filtrate laccase activity.

### 2.6. Composition Analysis

After harvesting the fruiting body, different samples were dried to constant weight at 60 °C in an oven and sealed for storage. Total protein (%), ash (%) and fat (%) were measured from this powder as previously described [9,31,32]. The total polysaccharides content was determined by the sulphuric acid anthrone colorimetric method [33]. The macro Kjeldahl method was used to measure the crude protein content (N× 4.38) of the mushrooms [34]. The crude fat content was determined by Soxhlet extraction (GB5009.6) [35], The standard procedure GB5009.4 [36] was used to measure their ash content in the fruiting body, All analyses were performed by PONY Testing International Group (Beijing, China) [9].

### 2.7. Statistical Analysis

Statistical analysis was performed with reference to Zhou et al. [9].

## 3. Results

### 3.1. Growth Rate of the Mycelia

**Figure 1:**
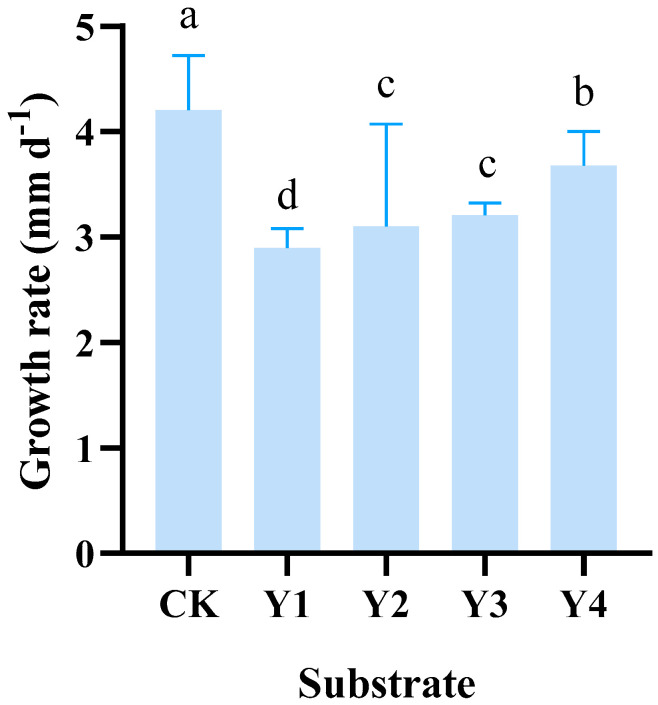
Mycelial growth rate of *P. eryngii* cultivated in different substrates. Different lowercase letters denote significant differences in each column (*p* < 0.05 according to Duncan’s test).

The duration of mycelial growth varied significantly under the different treatments as shown in Figure 1. The mycelial growth rate of *P. eryngii* on Y4 was 3.67 ± 0.35 mm d^−1^, which was lower than that of CK (4.21 ± 0.29 mm d^−1^) but higher than the rest treatment, while the growth rate of mycelia when treated with Y3 and Y2 were 3.21 ± 0.10 mm d^−1^ and 3.10 ± 0.39 mm d^−1^, respectively, which was slower than that of the traditional culture medium (CK). In addition, the growth rate of mycelia on Y1 was 2.90 ± 0.16 mm d^−1^, which was significantly lower than that of CK.

### 3.2. The Morphology and Characteristics of Fruiting Body of P. eryngii

The morphology and characteristics of the fruiting bodies grown on different substrates are shown in Table 2. The days of primordia to fruiting body from Y1 and Y2 were 17.8 and 17.9 days, respectively, which were longer than that of the CK (16.8 days). But under the conditions of Y3 and Y4, the days of primordia to fruiting body were 17.3 days, respectively, which was significantly different to CK. The fruiting body yields of Y2, Y3 and Y4 were 271.2 ± 26.0 g/bag, 273.2 ± 39.5 g/bag, and 267.1 ± 27.1 g/bag, respectively, which did not differ significantly from the CK (259.3 ± 37.4 g/bag). However, Y1 (303.1 ± 31.9 g/bag) was significantly higher than CK. The biological efficiency (BE) on different substrates ranged from 74.0% to 86.6%. Among them, Y1 was the most BE treatment as it was significantly higher than the CK. The weight of fruiting body and BE of the other treatment groups was not significantly different from that of the control group (*p* < 0.05).

The length of fruiting body and diameter of pileus of Y3 and Y4 treatments were significantly different from that of CK, while the length of fruiting body and diameter of pileus of Y1 and Y2 treatments were significantly lower than that of CK. The thickness of pileus of Y1, Y2, Y3 and Y4 treatments was basically the same as that of CK.

### 3.3. Nutrient Content of the Mushrooms

As shown in Table 3, the nutritional elements in *P. eryngii* cultured on different substrates varied significantly. The protein content of *P. eryngii* grown on substrate Y3 (treatment with 10.5% *G. biloba* leaf powder and sawdust and 21% sugarcane bagasse) was the highest, followed by substrate Y4 (treatment with 21% sawdust and 10.5% *G. biloba* leaf powder and sugarcane bagasse). While the protein content of *P. eryngii* grown on substrate Y1 (treatment with 21% *G. biloba* leaf powder and sugarcane bagasse) was slightly lower than that of substrate CK. The polysaccharide content of *P. eryngii* grown on substrate Y2 did not differ significantly from that of substrate CK, whereas *P. eryngii* grown in substrate Y4 was significantly lower than that of CK. The ash content of *P. eryngii* grown on substrate Y1, Y2, Y3 and Y4 was higher than that of CK, while the fiber content of Y1 and Y2 was higher than that of CK. The fat content of *P. eryngii* grown on Y1 substrate was higher than that of CK, while the fat content of *P. eryngii* grown on other substrates did not differ much from that of CK.

### 3.4. Laccase Activity of Mushrooms

As shown in Table 4, *P. eryngii* laccase activity varied considerably among different cultivation substrates cultivated at different periods. The laccase activity of *P. eryngii* cultivated on the five substrates was consistent throughout the growth process, with an increase at first and then a decrease. At the mycelia stage, Y4 (107.23 mU/mL) had the highest laccase activity, while Y1 had the highest laccase activity at all stages including complete mycelial colonization, young mushroom and fruiting body stage. At the time of full growth, the *P. eryngii* laccase activity of the five different substrates were the highest during the whole growth process, indicating that laccase had the strongest physical degradation ability at this time. Among them, Y1 (555.92 mU/mL) had the highest laccase activity, and Y3 (330.94 mU/mL) had the lowest laccase activity.

## 4. Discussion

**Figure 2:**
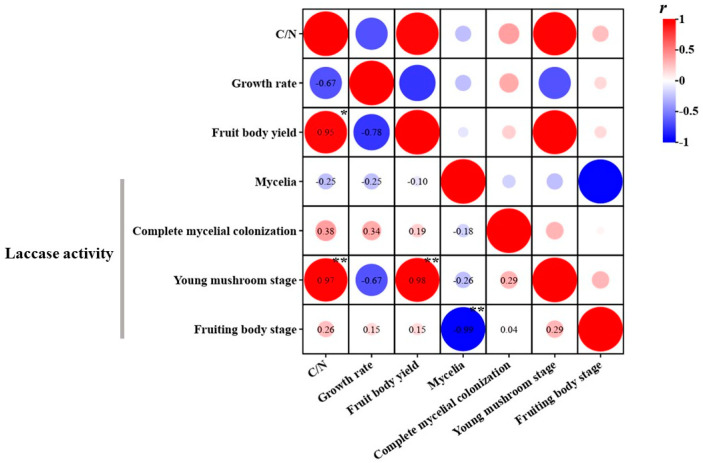
Effect of different substrates on the C:N rate, laccase activity and mycelial growth rate of *P. eryngii* (* indicates significance at *p* < 0.05; ** indicates significance at *p* < 0.01).

The C:N ratio is crucial for the growth of mushrooms as it significantly impacts both mycelium growth and fruiting bodies yield. Zhou et al. [37] investigated the effect of different C:N ratios on the growth of *P. eryngii* mycelium, and found that the most optimal ratio was between 20 and 25. When the C:N ratio becomes excessively high, it tends to restrain the growth of mycelium, which was also observed in this study. In this study, when the C:N ratio was increased from 25.28 to 28.58, mycelial growth slowed down by a maximum of 26.5%. The research conducted by Kurt et al. [38] revealed a negative correlation between the yield of *P. eryngii* and the C:N ratio. Contrary to their findings, our study presents an inconsistency, showing a progressive increase in yield as the C:N ratio rises (Figure 2). This may also be related to the physical structure of the substrate, as the medium containing 21% *G. biloba* leaf powder was more permeable. Variations in fungal mycelial growth and fruiting body yield are influenced not only by the C:N ratio but also by the physical property of the substrate utilized.

Studies have shown that the addition of different carbon and nitrogen sources affects mushroom yield. Among them, Wang et al. [39] increased the yields of *Pleurotus ostreatus* with the addition of wheat bran or soybean meal to the substrate, respectively, which resulted in increased yield on the basis of altered N source. Gao [40] increased the yield of *Pleurotus ostreatus* on the basis of replacing maize powder with 48% grapevine branches in the substrate, whereas when the carbon source sawdust or sugarcane bagasse was replaced by adding 10.5% or 21% of *G. biloba* leaf powder in this study, the yields of Y1–Y4 were 3.0–16.9% higher than the yields of CK (Table 2). This result suggested that the addition of *G. biloba* leaf powder can increase the yield of *P. eryngii*, which is consistent with the results of cultivating *P. eryngii* with korshinsk peashrub instead of wood chips and bagasse [32].

**Figure 3:**
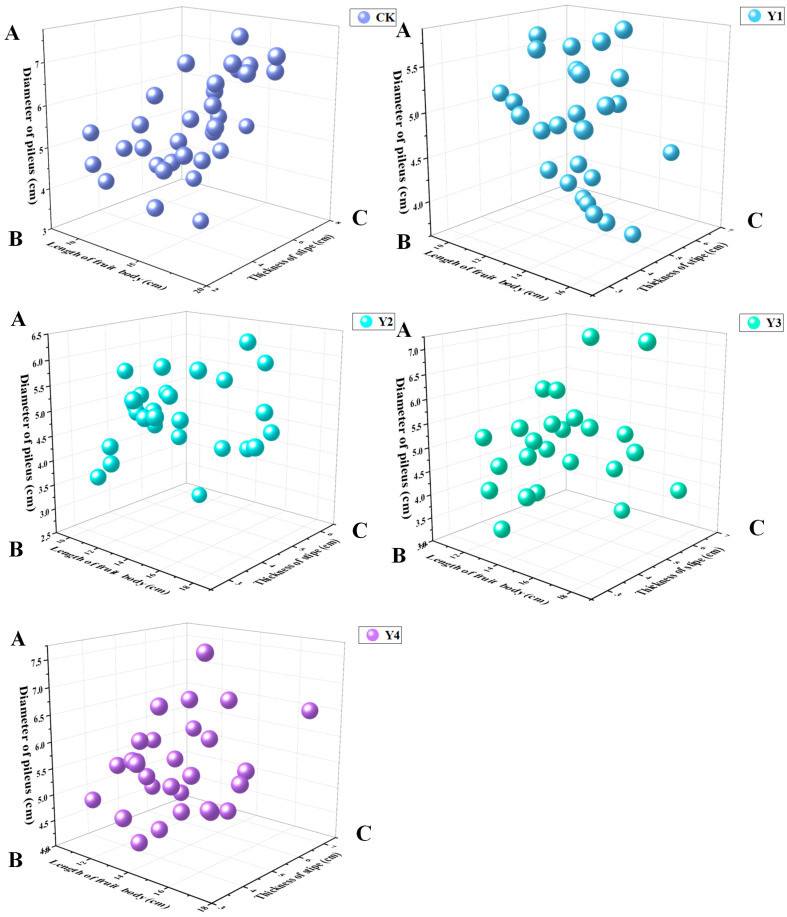
Scatter plot of the agronomic characteristics of fruiting body. A (*Y* axis): diameter of pileus (cm), B (*X* axis): length of fruiting body (cm), and C (*Z* axis): diameter of pileus (cm).

The agronomic traits of fruiting body of *P. eryngii* grown on *G. biloba* leaf powder substrate were not significantly different compared to CK (Table 2); the fruiting body length ranged from 13.4 to 14.1 cm. There were no significant differences in fruiting body length and stipe thickness in any of the treatment groups except for the substitution of 21% sawdust and sugarcane bagasse. The scatter plots of agronomic traits of fruiting body showed that mushrooms cultivated in Y1 and Y4 were more uniform in traits, while those cultivated in CK, Y2 and Y3 were more dispersed. Mushrooms of uniform size were more suitable for uniform collection, thus reducing labor costs (Figure 3). The combination of weight, shape and color is a good criterion for evaluating mushrooms [41]. Substrate Y1, Y2, Y3, and Y4 have the potential to be excellent media for growing *P. eryngii* which is rich in lignin and provides a beneficial carbon source. In the Chinese market, the price of sawdust is USD 97–110 per ton, and sugarcane bagasse is USD 138–152 per ton. Therefore, the effective use of *G. biloba* leaf greatly reduces the price of raw materials for cultivation and achieves energy saving and efficiency.

Numerous studies have shown that there are many factors that influence the content of mushroom proteins, polysaccharides, fats, and other nutrients. These factors include the type of substrate, the physical and biochemical properties of the substrate, and the type and amount of nutrients added to the substrate [9,32,42]. Zou et al. [32] increased the crude polysaccharide content by cultivating *P. eryngii* with different proportions of korshinsk peashrub as the substrate. In this study, the highest protein content (22.0%) was found in *P. eryngii* grown on 10.5% of *G. biloba* leaf powder and sawdust and 21% of sugarcane bagasse, whereas ash (5.82%) and fiber (7.57%) were found in *P. eryngii* grown on 21% of *G. biloba* leaf powder and sawdust substrate. This suggests that the use of *G. biloba* leaf powder is a new strategy for producing high quality *P. eryngii* to meet consumer demand.

Laccase is the main polyphenol oxidase involved in lignin degradation and studies have shown that it plays an important role in growth and development [43]. Laccase activity can be affected by various factors such as carbon and nitrogen sources, metal ions and activators, and pH [44]. Among the four stages, mycelia, complete mycelial colonization, young mushroom stage and fruiting body stage, complete mycelial colonization had the highest laccase activity. Substrate Y1 (treated with 21% *G. biloba* leaf powder and sugarcane bagasse) had the highest laccase activity at complete mycelial colonization compared to the other substrate (Table 4); it may secrete a substantial amount of laccase enzyme to break down the substrate, fulfilling its nutritional requirements of the growth and development of *P. eryngii*. Yet, as the fruiting body reaches maturity, the substrate becomes depleted, and the mycelium ages, resulting in a decreased production of the laccase enzyme. There was a significant positive correlation between the laccase activity of the young mushroom stage and the *P. eryngii* yield. However, mycelial growth rate was not positively correlated with the level of laccase activity, which is consistent with the results of a previous study by Zhao et al. [45].

## 5. Conclusions

This study confirmed the feasibility of using *G. biloba* leaf powder as a substrate for the cultivation of *P. eryngii* instead of sawdust and sugarcane bagasse in different proportions. Substrates containing 21% *G. biloba* leaf powder proved to be beneficial in increasing the mushroom yield, while the crude fiber content of the substrate was also higher. Therefore, *G. biloba* leaf powder can be used for commercial cultivation of *P. eryngii* to reduce the supply of sawdust and sugarcane bagasse. Thus, the problem of reduced supply and increased price of substrate components can be solved. These results suggest that mushroom growers can use a practical method to produce high yields of *P. eryngii*.

## Figures and Tables

**Table 1:** Composition of ingredients for the substrate with different inclusion rates for *P. eryngii* cultivation.

Sustrate	Sadust *	Sugarcane Bagasse *	*Ginkgo biloba* Leaf Powder *	Cottonseed Hull *	Ground Corncobs *	Wheat Bran *	Maize Powder *	Soybean Meal *	Lime *	Gypsum *	C:N
CK	21	21	nd	4.2	18.4	18.4	6.8	8.2	1	1	25.52
Y1	nd	21	21	4.2	18.4	18.4	6.8	8.2	1	1	28.58
Y2	21	nd	21	4.2	18.4	18.4	6.8	8.2	1	1	26.31
Y3	10.5	21	10.5	4.2	18.4	18.4	6.8	8.2	1	1	25.60
Y4	21	10.5	10.5	4.2	18.4	18.4	6.8	8.2	1	1	25.28

* indicates dry matter, g 100 g^−1^. nd: not detected.

**Table 2:** Agronomic traits of *P. eryngii* when cultivated on different substrates (mean ± SD, n = 30).

Substrate	Days of Primordia to Fruiting Body (d)	Fruiting Body Yield (g/bag)	Biological Efficiency (%)	Length of Fruiting Body (cm)	Thickness of Stipe (cm)	Diameter of Pileus (cm)
CK	16.8 ± 0.4 c	259.3 ± 37.4 b	74.0 ± 10.7 b	15.4 ± 2.0 a	4.6 ± 1.0 a	6.2 ± 1.2 a
Y1	17.8 ± 0.8 a	303.1 ± 31.9 a	86.6 ± 9.5 a	13.6 ± 1.3 c	4.5 ± 0.7 a	4.9 ± 0.7 c
Y2	17.9 ± 0.8 a	271.2 ± 26.0 b	77.4 ± 7.4 b	13.4 ± 2.2 c	4.2 ± 0.8 a	5.2 ± 0.8 c
Y3	17.3 ± 0.5 b	273.2 ± 39.5 b	78.0 ± 11.8 b	14.0 ± 1.7 b	4.5 ± 0.9 a	5.5 ± 1.0 bc
Y4	17.3 ± 0.5 b	267.1 ± 27.1 b	76.3 ± 7.7 b	14.1 ± 1.8 b	4.7 ± 0.8 a	5.4 ± 0.8 bc

Note: Different lowercase letters denote significant differences in each column. Different letters indicate significant differences between the strains (*p* < 0.05, according to Tukey’s test). CK: treatment with 21% sawdust and sugarcane bagasse. Y1: treatment with 21% *G. biloba* leaf powder and sugarcane bagasse. Y2: treatment with 21% *G. biloba* leaf powder and sawdust. Y3: treatment with 10.5% *G. biloba* leaf powder and sawdust and 21% sugarcane bagasse. Y4: treatment with 21% sawdust and 10.5% *G. biloba* leaf powder and sugarcane bagasse.

**Table 3:** Nutritional value of *P. eryngii* when cultivated on different substrates (100 g^−1^, mean ± SD, n = 3).

Substrate	Protein (g)	Ash (g)	Fiber (g)	Fat (g)	Polysaccharide (g)
CK	18.3 ± 0.00 d	5.12 ± 0.07 d	7.37 ± 0.21 c	1.03 ± 0.01 b	4.11 ± 0.07 a
Y1	17.6 ± 0.00 e	5.60 ± 0.07 c	7.43 ± 0.12 b	1.11 ± 0.01 a	3.91 ± 0.03 b
Y2	18.4 ± 0.00 c	5.82 ± 0.05 a	7.57 ± 0.15 a	1.03 ± 0.01 b	4.11 ± 0.12 a
Y3	22.0 ± 0.06 a	5.72 ± 0.04 b	7.23 ± 0.06 d	1.01 ± 0.02 b	3.87 ± 0.06 b
Y4	19.7 ± 0.00 b	5.52 ± 0.04 c	7.23 ± 0.15 d	1.01 ± 0.01 b	2.85 ± 0.07 c

Note: Different lowercase letters denote significant differences in each column. Different letters indicate significant differences between the strains (*p* < 0.05, according to Tukey’s test). CK: treatment with 21% sawdust and sugarcane bagasse. Y1: treatment with 21% *G. biloba* leaf powder and sugarcane bagasse. Y2: treatment with 21% *G. biloba* leaf powder and sawdust. Y3: treatment with 10.5% *G. biloba* leaf powder and sawdust and 21% sugarcane bagasse. Y4: treatment with 21% sawdust and 10.5% *G. biloba* leaf powder and sugarcane bagasse.

**Table 4:** Laccase activity in different substrates (mean ± SD, n = 3).

Substrate	Laccase Activity (mU/mL)			
	Mycelia Stage	Complete Mycelial Colonization Stage	Young Mushroom Stage	Fruiting Body Stage
CK	104.38 ± 0.29 d	550.82 ± 12.75 a	10.97 ± 1.31 b	10.54 ± 1.45 a
Y1	105.21 ± 0.63 c	555.92 ± 36.43 a	18.02 ± 4.05 a	8.55 ± 3.39 a
Y2	106.39 ± 0.40 b	447.95 ± 27.26 bc	11.82 ± 2.36 b	4.70 ± 0.30 b
Y3	105.49 ± 0.53 c	330.94 ± 49.37 d	12.28 ± 1.07 b	8.57 ± 1.00 a
Y4	107.23 ± 0.49 a	511.36 ± 41.79 ab	11.10 ± 1.22 b	1.90 ± 2.12 b

Note: Different lowercase letters denote significant differences in each column. Different letters indicate significant differences between the strains (*p* < 0.05, according to Tukey’s test). CK: treatment with 21% sawdust and sugarcane bagasse. Y1: treatment with 21% *G. biloba* leaf powder and sugarcane bagasse. Y2: treatment with 21% *G. biloba* leaf powder and sawdust. Y3: treatment with 10.5% *G. biloba* leaf powder and sawdust and 21% sugarcane bagasse. Y4: treatment with 21% sawdust and 10.5% *G. biloba* leaf powder and sugarcane bagasse.

## Data Availability

The data used to support the study findings are included within the article.
